# Chickpea attenuates postprandial blood glucose responses: a systematic review and meta-analysis

**DOI:** 10.1186/s12937-025-01176-8

**Published:** 2025-07-14

**Authors:** Eunice Mah, Cassi N. Uffelman, Traci M. Blonquist, Ding Ding Wang, Colin D. Rehm, Shellen R. Goltz, YiFang Chu

**Affiliations:** 1 Biofortis, Inc, Addison, IL USA; 2 D&V Systematic Evidence Review Consulting, LLC, La Jolla, CA USA; 3 PepsiCo R&D Life Sciences, Purchase, NY USA; 4 PepsiCo R&D Life Sciences, 433 W Van Buren St, Chicago, IL 60607 USA

**Keywords:** Garbanzo, Cicer arietinum, Blood sugar, Legumes, Healthy adults

## Abstract

**Background:**

Chickpeas are a legume that may help improve glycemic control, but their acute effects on postprandial glucose and insulin responses are unclear. This systematic review and meta-analysis aimed to assess the impact of acute chickpea consumption on these outcomes in controlled, crossover trials.

**Methods:**

We screened PubMed, Cochrane Central Register of Controlled Trials (CENTRAL), and Embase from inception through March 21, 2024 for acute, controlled, experimental (randomized or non-randomized) trials comparing chickpea consumption with carbohydrate-matched controls that reported on postprandial glucose and insulin responses (iAUC and C_max_). Two reviewers extracted the data and assessed risk-of-bias (RoB 2) and certainty-of-evidence (GRADE). Data were analyzed using generic inverse-variance with random-effects model.

**Results:**

A total of 28 eligible studies (40 comparisons) were identified. Chickpea consumption significantly reduced postprandial glucose iAUC compared to carbohydrate-matched controls (MD: -47.89, 95% CI: -64.20, -31.58, *p* < 0.0001). No significant effects were observed on glucose C_max_ (MD: -0.23, 95% CI: -1.48, 1.02, *p* = 0.7207) or insulin iAUC (MD: 50.06, 95% CI: -3771.14, 3871.26, *p* = 0.9795). The GRADE assessment indicated very low certainty for glucose iAUC due to heterogeneity.

**Conclusion:**

Meta-analysis of controlled trials suggest that acute chickpea consumption lowers postprandial glucose iAUC, albeit with low certainty of evidence. While no significant effects were observed on glucose peak or insulin response, the findings align with previous research on pulses and glycemic control. Further high-quality studies are needed to confirm these findings, as the current evidence is of low to very low certainty. Future studies should explore the long-term effects of chickpea consumption, investigate the impact of processing methods, and include metabolically unhealthy populations to enhance generalizability.

**Registration:**

This review was registered on PROSPERO (CRD42022365074).

**Supplementary Information:**

The online version contains supplementary material available at 10.1186/s12937-025-01176-8.

## Background

It is well established that type 2 diabetes mellitus (T2DM) is a major public health concern. Importantly, having T2DM is a risk factor for developing cardiovascular disease (CVD) [1], which together represent two of the top 10 leading causes of death in the United States of America (USA) and worldwide [2, 3]. Given the profound impact of T2DM and CVD, effective prevention and management strategies are critical. Among modifiable factors, diet plays a pivotal role in controlling blood glucose levels and reducing the risk of these chronic diseases. In particular, low glycemic index (GI) diets have been shown to help manage postprandial glucose responses and lower the risk of T2DM and CVD [4–6].

Pulses—the edible seeds of legumes, such as beans, peas, lentils, and chickpeas—are emerging as important dietary components for glycemic control and overall health. These nutrient-dense foods are low in energy, high in fiber, and rich in essential nutrients like iron, zinc, and potassium [7, 8]. Pulses are known for their low glycemic index, meaning they cause smaller spikes in blood glucose levels compared to other carbohydrate sources [9–12]. As such, they are gaining attention for their potential role in the prevention and management of T2DM and CVD and are recommended by various diabetes associations worldwide as a means of optimizing diabetes control through lowering the GI and increasing the dietary fiber content of the diet [13–15]. Among pulses, chickpeas are particularly notable for their favorable glycemic properties [16]. Research indicates that chickpeas elicit amongst the lowest glycemic responses compared to other pulses [9–12]. In addition to their low GI, chickpeas contain bioactive compounds with putative anti-diabetic and anti-obesity properties [17, 18], which may further enhance their value in preventing T2DM and CVD. Despite these promising attributes, evidence on the effects of pulses—and specifically chickpeas—on glycemic control remains inconsistent across studies [19–22].

Previous meta-analyses of observational studies found no clear association between pulse intake and the incidence of T2DM, but pulse consumption is typically low in the general population, making it difficult to detect associations [19, 20]. However, experimental studies suggest that pulse consumption may improve glycemic control markers, such as fasting blood glucose and HbA1c [21, 22]. These conflicting findings highlight the need for more targeted research on specific pulses like chickpeas.

The purpose of this systematic review and meta-analysis is to assess the effects of acute chickpea consumption on postprandial glucose and insulin responses in adults using data from peer-reviewed experimental trials. During the conduct of this work, a meta-analysis was published in 2023 and concluded that chickpea consumption reduces blood glucose when compared to wheat and potatoes [16]. This meta-analysis focused on comparing chickpeas with specific types of carbohydrate-rich comparators (e.g., wheat, potatoes, pasta) and did not account for variations in available carbohydrate (avCHO) content, which can independently affect glycemic responses. Our review addresses this gap by comparing chickpea interventions with controls that contain equivalent amounts of avCHO. This approach isolates the intrinsic glucoregulatory effects of chickpeas and aligns with the USA FDA’s recommendations for evaluating the physiological effects of dietary fiber [23]. By refining the methodology and addressing limitations in prior research, this work offers updated insights that may help inform dietary recommendations for individuals with or at risk of T2DM.

## Methods

This systematic review and meta-analysis was conducted according to the Cochrane Handbook for Systematic Reviews of Interventions [24]. Data were reported in accordance with the Preferred Reporting Items for Systematic Reviews and Meta-Analyses (PRISMA) guidelines [25]. This review was registered on PROSPERO (CRD42024538112).

### Inclusion and exclusion criteria

The Population, Intervention, Comparison, Outcome, and Study design (PICOS) criteria defining our research question are presented in Table 1.

**Table 1:** Description of the research question and PICOS framework

Parameter	Description
Population	Non-pregnant and non-lactating humans of any health status or age
Intervention	Groups consuming chickpeas of any form or amount (e.g., whole, pureed, hummus, products containing chickpea flour/powder)
Control	Groups consuming a carbohydrate-containing meal without chickpeas
Outcome	Postprandial glucose and insulin responses Primary: glucose iAUC (incremental area under the curve) Secondary: glucose maximum concentration (C_max_), insulin iAUC, insulin C_max_
Study Design	Peer-reviewed, acute, controlled, experimental (randomized or non-randomized) trials
Research Question	What is the effect of acute chickpea consumption on postprandial glucose and insulin responses?

Inclusion criteria were: English language; non-pregnant and non-lactating humans of any health status or age; chickpea consumption of any form or amount (e.g., whole, pureed, hummus, products containing chickpea flour/powder); carbohydrate-containing control group without chickpeas; reporting on at least one postprandial outcome of interest; peer-reviewed, acute, controlled, experimental (randomized or non-randomized) trials.

Exclusion criteria were: not available in English; study populations including pregnant or lactating humans; no reported chickpea consumption or consumption of mixed meals in which the independent effects of chickpeas cannot be isolated; no outcomes of interest reported or not available (i.e., not available from authors following at least three attempts to contact and no figures available in the publication for extraction); single-arm studies, chronic interventions, observational, animal, or cell model studies; full article not available; not original research (review articles, commentaries, grey literature).

To reduce heterogeneity among studies included in the meta-analysis, additional criteria for inclusion were: control group containing an approximately equivalent amount of avCHO and postprandial outcomes reported over 120 min [23, 26]. Exclusion criteria were: avCHO not reported or not approximately equal between the intervention and control groups; postprandial glucose or insulin iAUC not reported over 120 min (e.g., over 180 min). Finally, a minimum of three studies per outcome were required to be included in the quantitative meta-analysis and 10 studies per outcome were required for assessment of potential publication bias.

### Search strategy

A search of three databases including PubMed, Cochrane Central Register of Controlled Trials (CENTRAL), and Embase was conducted from inception through March 21, 2024. Electronic searches were supplemented with manual searches of references from included articles along with systematic reviews and meta-analyses [16, 22, 27] identified during our search. The detailed search strategy is outlined in Supplemental Table 1.

### Article screening and data extraction

Two investigators (CNU and DDW) independently screened articles to determine their eligibility through a two-step process: (1) potential eligibility based on the title and abstract and (2) confirmation of eligibility based on a full-text assessment of qualified abstracts.

Upon confirmation of eligibility, investigators (CNU and DDW) independently reviewed and extracted relevant data from each included study using a data extraction template. Extracted data included study design, location, eligibility criteria, participant characteristics (e.g., health status, age, sex, BMI), intervention and comparator details (e.g., amount, duration, nature of foods [e.g. glucose, bread, pasta], available carbohydrate), outcome measurement (e.g., type, matrix, timepoints, analytical method), confounders and effect modifiers that were adjusted for in the statistical analysis, and results. In the absence of numerical values for outcome data and the inability to contact study authors after three attempts, values were extracted from figures using Plot Digitizer, version 3.1.5 (Free Software Foundation, Boston, MA) if relevant figures were included in the publication. Any discrepancies in the screening and data extraction process were discussed until a consensus was reached.

### Risk of bias assessment

Risk of bias was independently evaluated by the same two investigators (CNU and DDW) using version 2 of the Cochrane risk-of-bias (RoB 2) tool [28]. Bias was assessed in five distinct domains: (1) bias arising from the randomization process, (2) bias due to deviations from intended interventions, (3) bias due to missing outcome data, (4) bias in the measurement of the outcome, and (5) bias in the selection of the reported result. Within each domain, the investigators answered one or more signaling questions and these answers led to judgments of “low risk of bias”, “some concerns”, or “high risk of bias”. Any discrepancies in the risk of bias assessments were graded by a third investigator (EM) and discussed as a group until a consensus was reached.

### Analysis

#### Qualitative analysis

Articles that reported postprandial glucose or insulin outcomes but did not assess iAUC over 120 min or did not ensure approximately equal avCHO between chickpea and comparator groups were deemed ineligible for the meta-analysis. These studies were included in a qualitative summary of results instead. An effect direction plot was used to assess the impact of chickpea consumption on postprandial outcomes compared to the comparator group [29]. To draw conclusions for each outcome, a minimum of three comparisons was required. When fewer than three comparisons were available, evidence was considered insufficient. Conclusive statements were based on the following criteria: a clear majority (≥ 70%) of comparisons needed to report the same direction of effect to support a finding. If fewer than 70% of comparisons reported the same direction of effect, results were deemed inconsistent or indicative of no clear effect.

#### Quantitative analysis

Numeric values reported in the original manuscripts were converted as follows: standard error was converted to standard deviation (SD $\:=$ SE $\:\times\:\surd\:N$), glucose concentrations reported in mg/dL were converted to SI units (mmol/L $\:=$ mg/dL $\:\times\:$ 0.0555), and insulin concentrations reported in µIU/mL were converted to SI units (pmol/L$\:=$ µIU/mL $\:\times\:$ 6.945). Means were rounded to the nearest tenth and the standard deviation was rounded to the nearest hundredth value.

The meta-analyses were conducted using the R packages “metafor” (Version 4.6-0) and “meta” (Version 8.0–1) [30]. These packages were used to estimate the random-effects model, assess heterogeneity, generate funnel plots, and conduct Egger’s bias test. The generic inverse-variance approach was applied. For crossover studies with multiple chickpea intervention arms, the means and SD for glucose and insulin iAUC and/or C_max_ at the end of each chickpea intervention were combined according to the Cochrane Handbook (Sect. 6.5.2.10) [24]. The standard error of the standardized mean difference (SMD) was calculated using an imputed correlation value of 0.50 to provide a conservative estimate based on the assumption that this value would minimize the error of effect-size estimates. There were no qualitative differences when the outcomes were combined using mean differences (MDs) or SMDs; thus, MDs were reported for all outcomes as this is more intuitive and easier to be reviewed for practical significance.

Inter-study heterogeneity was assessed using the Cochran Q statistic and quantified using the I^2^ statistic, where I^2^ ≥ 50% and *P* < 0.10 were considered evidence of substantial heterogeneity [24]. Outliers were identified as studies with 95% confidence interval (CI) that did not overlap with the pooled effect 95% CI [31]. Analyses were performed with and without the removal of outliers.

Publication bias was evaluated through visual inspection of funnel plots for asymmetry and formal testing using Egger’s test for outcomes with at least 10 comparisons [32, 33]. To avoid inflation of false positive results [34], the standard error was modified as suggested [35, 36].

We intended to perform subgroup analysis such as by health status, form of chickpea, or study quality. However, there was insufficient data per subgroup to complete any meaningful quantitative analyses.

The certainty of the evidence was assessed using the GRADE (Grading of Recommendations, Assessment, Development, and Evaluation) framework using GRADEpro GDT (McMaster University and Evidence Prime), which assigns a grade of high, moderate, low, or very low certainty [36]. Randomized controlled trials were initially considered high-certainty evidence but may be downgraded based on pre-specified criteria including risk of bias, inconsistency, indirectness, imprecision, and publication bias.

## Results

### Search results

**Fig. 1:**
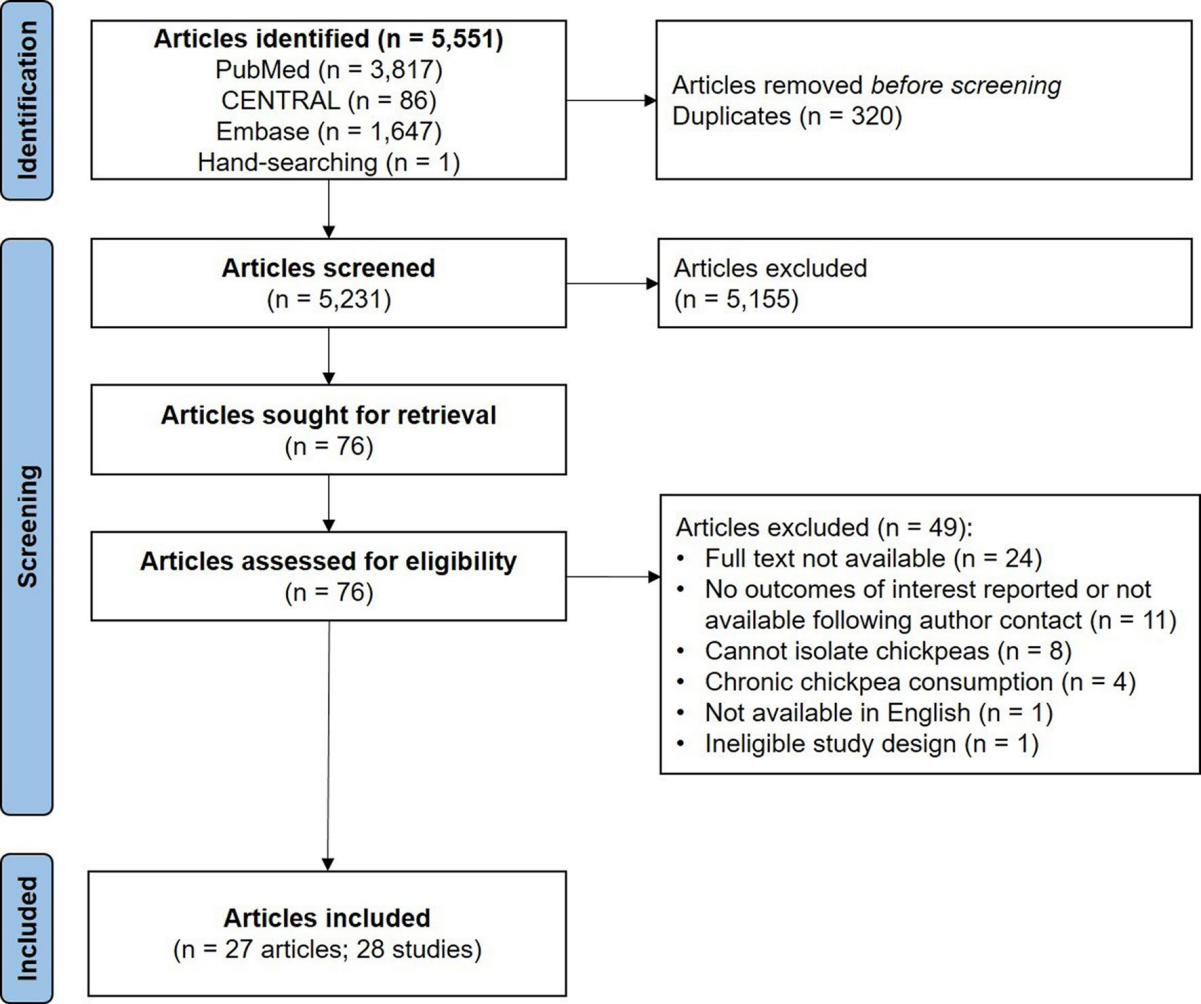
PRISMA flow diagram of the article identification, screening, and inclusion process

A total of 5,551 articles were identified. After removing 320 duplicates, 5,231 articles remained for screening. During the initial title and abstract screening, 5,155 articles were excluded for not meeting the inclusion criteria. In the full-text screening of 76 articles, 49 were excluded for various reasons. Ultimately, 27 articles comprising 28 studies were eligible for inclusion (Fig. 1**)**.

### Study characteristics

The 28 studies (40 comparisons) were all acute, experimental crossover trials. Of these, 25 were randomized controlled trials, one was a balanced-order incomplete block design [37], one was semi-randomized [38], and one was a non-randomized crossover trial [39]. Sample sizes ranged from 10 to 38 participants, who were generally healthy (except for one study that included exclusively individuals with T2DM and another that included individuals with and without T2DM). Across all studies, participants’ ages ranged from 21.3 to 53 years, and BMI means ranged from 21.6 to 29.4 kg/m². Of the 28 studies, 16 included both male and female participants, five included only males, five included only females, and two did not report participant sex. Study locations included: Canada (*n* = 8), the United Kingdom (UK; *n* = 5), Kuwait (*n* = 4), Australia (*n* = 2), the USA (*n* = 2), and one study each from the Netherlands, Lebanon, Pakistan, the Philippines, New Zealand, Greece, and Sri Lanka.

Most studies assessed the effects of consuming chickpea powder/flour (*n* = 19 subgroups) or boiled/canned whole chickpeas (*n* = 16 subgroups). Other chickpea forms included pureed chickpeas (*n* = 2), hummus (*n* = 1), and chickpea salad dip (*n* = 1). One study did not report the chickpea form. Comparators (*n* = 29) were primarily high-glycemic foods, including white bread (*n* = 15) and wheat bread (*n* = 5). Other comparators included brown rice (*n* = 2), macaroni and cheese (*n* = 2), potato (*n* = 2), white rice (*n* = 1), a dextrose beverage (*n* = 1), and an extruded corn snack (*n* = 1). Available carbohydrate amounts for chickpea and comparator groups ranged from 12.9 to 100.4 g.

Postprandial outcomes were measured using fingerstick glucometers, intravenous catheters, or continuous glucose monitors (CGM). Biological matrices used for assessment included whole blood, plasma, serum, capillary blood, and interstitial glucose. Study characteristics, including author, year, design, sample size, interventions, and outcomes, are summarized in Table 2. The number of articles and comparisons included by outcome is presented in Table 3.

**Table 2:** Study characteristics

Study, Year	Study Type and Design	Location	Sample Size (Sex)	Health Status	Age, years (SD)	BMI, kg/m^2^ (SD)	Chickpea Intervention			Comparator		Outcomes Assessed, Matrix, and Method
							Amount, g	avCHO, g	Description	avCHO, g	Description	
Anderson 2014 [50]	RCT, crossover	Canada	12 (12 M)	Healthy	23.6 (3.5)	22.3 (1.4)	25 g cooked	38.7	Whole canned chickpeas + tomato sauce	38.7	Whole-wheat flour + tomato sauce	Glucose iAUC
									Pureed canned chickpeas + tomato sauce			Whole blood
									Canned chickpea, powder + tomato sauce			Fingerstick
Atkinson 2021 [59]	RCT, crossover	Australia	12 (4 M, 8 F)	Healthy	24.9 (20.4)	23.1 (10)	131.5 g	25	Canned chickpeas	25	White bread	Glucose iAUC
												Plasma
												Fingerstick
Augustin 2016 [51]	RCT, crossover	Canada	10 (7 M, 3 F)	Healthy	53 (7)	29.4 (12)	259 g	25	Hummus	25	White bread	Glucose iAUC Insulin iAUC
												Plasma; Serum
												Fingerstick
Bajka 2021 (Study 1) [52]	RCT, crossover	UK	20 (8 M, 12 F)	Healthy	26 (4.08)	23.8 (4.67)	100 g	58	Beverage made from chickpea intact cell powder (ICP) + Nesquick chocolate powder	58	Beverage made from dextrose (Nesquick chocolate powder)	Glucose iAUC Glucose C_max_
												Interstitial glucose and capillary blood glucose
									Beverage made from chickpea ruptured cell powder (RCP) + Nesquick chocolate powder			
												CGM and fingerstick
Bajka 2021 (Study 2) [52]	RCT, crossover	UK	21 (10 M, 10 F)*	Healthy	27.9 (4.91)	24.7 (3.1)	NR	48.3	Bread containing 30% w/w chickpea intact cell powder (ICP) + 20 g of no-added sugar strawberry jam	48.1	100% whole wheat bread + 20 g of no-added sugar strawberry jam	Glucose iAUC Glucose C_max_
												Interstitial glucose
								48.3	Bread containing 60% w/w chickpea intact cell powder (ICP) + 20 g of no-added sugar strawberry jam			
												CGM
Bajka 2023 [49]	RCT, crossover	UK	20 (10 M, 10 F)	Healthy	NR; inclusion range 18–45	NR; inclusion range 18.0–35.0	NR	48.3	Bread containing 30% w/w cellular chickpea powder (CCP) + 20 g of no-added sugar strawberry jam	48.1	100% white flour bread + 20 g of no-added sugar strawberry jam	Glucose iAUC Glucose C_max_ Insulin iAUC Insulin C_max_
												Plasma
								48.2	Bread containing 60% w/w cellular chickpea powder (CCP) + 20 g of no-added sugar strawberry jam			
												Venous cannula
Boers 2016 [37]	RCT, crossover (balanced-order incomplete block)	UK	38 (3 M, 35 F)	Healthy	37 (9)	22.8 (1.6)	15 g chickpea flour	57	Bread containing 15 g chickpea flour + 85 g high-fiber wheat flour	61	Bread containing 100% high-fiber wheat flour	Glucose iAUC Glucose C_max_
												Plasma
												Fingerstick
Chen 2022 [38]	Semi-randomized crossover	Netherlands	26 (NR)	Healthy	NR; inclusion range 18–55	NR	323 g	51	Boiled chickpeas chewed for 37 s (long chewing time)	63	Brown rice chewed for 41 s (long chewing time)	Glucose iAUC Glucose C_max_
												Whole blood
									Boiled chickpeas chewed for 20 s (short chewing time)		Brown rice chewed for 23 s (short chewing time)	
												CGM
Dandachy 2018 [40]	RCT, crossover	Lebanon	16 (16 F)	Healthy	22.9 (12)	22.7 (11)	NR	NR	Chickpeas mankoushe (mixture of 70% refined wheat and 30% pre-processed chickpea flour)	NR	Regular mankoushe (100% refined wheat flour)	Glucose iAUC Insulin iAUC
												Serum Plasma
												Catheter
Hafiz 2022 [53]	RCT, crossover	UK	13 (4 M, 9 F)	Healthy	28.7 (6.6)	23.2 (11)	250 g cooked	50	Whole canned chickpeas	50	Mashed potatoes	Glucose iAUC Glucose C_max_
							250 g cooked		Pureed canned chickpeas			Interstitial glucose
							NR; 217 g pasta		Chickpea pasta made from chickpea flour			CGM and venous catheter
Johnson 2005 [54]	RCT, crossover	Australia	11 (9 M, 2 F)	Healthy	32 (6.6)	24.7 (2.7)	NR; 401 g bread	50	Chickpea bread (24.3% chickpea flour, 75.7% wheat flour) + margarine + jam	50	White bread (100% wheat flour) + margarine + jam	Glucose iAUC Insulin iAUC
												Plasma Serum
									Extruded chickpea bread (24.3% chickpea flour, 75.7% wheat flour) + margarine + jam			
												Catheter
Johnston 2021 [9]	RCT, crossover	Canada	26 (14 M, 12 F)	Healthy	24.7 (29.1)	23.5 (12)	NR; 50 g serving	NR	Extruded corn snack with 40% pulse flour from chickpea	NR	Extruded corn snack with 100% flour from corn	Glucose iAUC Insulin iAUC
												Serum
												Intravenous catheter
Khawaja 2012 [39]	Non-randomized crossover	Pakistan	12 (8 M, 4 F)	Healthy	25.7 (7.5)	23.2 (4.8)	NR	50.2	Chickpea flour chapatti	50	White bread	Glucose iAUC
												Whole blood
			10 (7 M, 3 F)	T2DM	47.9 (7.7)	27.4 (11)						
												Fingerstick
Mehio 1997 [41]	RCT, crossover	USA	12 (5 M, 7 F)	Healthy	24 (3.4)	22.8 (2.1)	237 g	26	Chickpea salad dip	48.6	White bread	Glucose iAUC Insulin iAUC
												Serum
												Intravenous catheter
Mollard 2011 [43]	RCT, crossover	Canada	25 (25 M)	Healthy	21.3 (2.5)	21.6 (1.5)	222.8 g	98.7	Canned chickpeas + pasta + tomato sauce	100.4	Macaroni and cheese	Glucose iAUC
												Whole blood
												Fingerstick
Mollard 2012 [42]	RCT, crossover	Canada	24 (24 M)	Healthy	24.3 (17.6)	22.8 (6.9)	Ad libitum	12 g per 100 g serving	Chickpeas + pasta + tomato sauce (homogenized)	100.4	Macaroni pasta + tomato sauce (homogenized)	Glucose iAUC
												Whole blood
												Fingerstick
Mollard 2014 [10]	RCT, crossover	Canada	15 (15 M)	Healthy	22.5 (3.1)	22.9 (1.5)	222.8 g	47.8	Canned chickpeas + tomato sauce	64	White bread	Glucose iAUC
												Whole blood
												Fingerstick
Panlasigui 1995 [44]	RCT, crossover	Philippines	11 (5 M, 6 F)	Healthy	22 (3.6)	NR	100 g dry	50	Boiled chickpeas	50	White bread	Glucose iAUC
												Whole blood
												Fingerstick
Venn 2006 [55]	RCT, crossover	New Zealand	20 (9 M, 11 F)	Healthy	23.3 (3.5)	23.4 (3.3)	70 g cooked	12.9	Chickpeas (form not reported)	15.7	White bread	Glucose iAUC
												Whole blood
												Fingerstick
Voyatzoglou 1995 [45]	RCT, crossover	Greece	10 (3 M, 7 F)	T2DM	NR	NR; range 25.53–35.95	NR	NR; 50 g total CHO	Boiled chickpeas + olive oil	NR; 50 g total CHO	White bread	Glucose iAUC Insulin iAUC
												Whole blood
												Fingerstick
Widanagamage 2009 [11]	RCT, crossover	Sri Lanka	10 (NR)	Healthy	NR; range 20–30	23 (3.2)	200 g cooked	25 g digest-ible starch	Boiled chickpeas + coconut oil	25 g digest-ible starch	White bread	Glucose iAUC
												Serum
												Fingerstick
Winham 2017 [56]	RCT, crossover	USA	12 (12 F)	Healthy	36 (13.9)	23.3 (3.1)	130 g cooked	47.6	Canned chickpeas + plain white rice	49.5	Plain white rice	Glucose iAUC Insulin iAUC
												Plasma
												NR
Wong 2009 [12]	RCT, crossover	Canada	15 (15 M)	Healthy	NR; inclusion range 18–35	NR; inclusion range 20–25	341 g cooked	47.8	Canned chickpeas + tomato sauce	64	White bread + tomato sauce	Glucose iAUC
												Whole blood
												Fingerstick
Zafar 2015 [46]	RCT, crossover	Kuwait	13 (13 F)	Healthy	21.4 (8.3)	23.6 (8.7)	NR; 50 g serving	NR	Whole wheat bread made with 25% chickpea flour	NR	Whole wheat bread	Glucose iAUC Glucose C_max_
												Whole blood
									Whole wheat bread made with 35% chickpea flour			
												Fingerstick
Zafar 2017 (ref)	RCT, crossover	Kuwait	12 (12 F)	Healthy	23.67 (6.5)	22.39 (4.6)	200 g cooked	18	Canned chickpeas	18	White bread + butter	Glucose iAUC
												Whole blood
												Fingerstick
Zafar 2019 [6)	RCT, crossover	Kuwait	14 (14 F)	Healthy	NR; inclusion range 17–30	NR; inclusion range 20–25	342 g	50	Canned chickpeas + butter	50	White bread + butter	Glucose iAUC
												Whole blood
												Fingerstick
Zafar 2020 [47]	RCT, crossover	Kuwait	15 (NR; M and F included)	Healthy	22.4 (2.4)	22.8 (2.2)	NR	50	White bread substituted with 20% chickpea flour	50	White bread (100% white flour)	Glucose iAUC
									White bread substituted with 30% chickpea flour			Whole blood
									White bread substituted with 40% chickpea flour			Fingerstick
Zurbau 2018 [58]	RCT, crossover	Canada	17 (8 M, 9 F)	Healthy	26.7 (12.3)	22.2 (2.8)	113 g dry	50	Boiled chickpea + fresh tomatoes + extra virgin olive oil	50	Large potato + cheese alternative + fresh tomatoes + extra virgin olive oil	Glucose iAUC
												Whole blood
												Fingerstick

Data for age and BMI are mean (SD) unless otherwise noted

Separate rows within the chickpea intervention denote different intervention arms

* Authors reported 21 participants enrolled, but table of characteristics only provided data for 20 subjects (i.e., 10 M, 10 F)

Abbreviations: avCHO, available carbohydrate; CCP, cellular chickpea powder; CGM, continuous glucose monitor; F, females; iAUC, incremental area under the curve; ICP, intact cell powder; M, males; NR, not reported; RCP, ruptured cell powder; RCT, randomized controlled trial

**Table 3:** Summary of studies included in the systematic review and meta-analysis by outcome

Parameter	Systematic Review			Meta-analysis		
	# of Studies	# of Comparisons	References	# of Studies	# of Comparisons	References
Glucose iAUC	13	18	Chen 2022 Dandachy 2018 Johnston 2021 Khawaja 2012 Mehio 1997 Mollard 2011 Mollard 2012 Mollard 2014 Panlasiagui 1995 Voyatzoglou 1995 Wong 2009 Zafar 2015 Zafar 2020	15	23	Anderson 2014 Atkinson 2016 Augustin 2016 Bajka S1 2021 Bajka S2 2021 Bajka 2023 Boers 2016 Hafiz 2022 Johnson 2005 Venn 2006 Widanagamage 2009 Winham 2017 Zafar 2017 Zafar 2019 Zurbau 2019
Glucose Peak	2	4	Chen 2022 Zafar 2015	5	8	Bajka S1 2021 Bajka S2 2021 Bajka 2023 Hafiz 2022
Insulin iAUC	4	4	Dandachy 2018 Johnston 2021 Mehio 1997 Voyatoglou 1995	4	6	Augustin 2016 Bajka 2023 Johnson 2005 Winham 2017
Insulin Peak	1	2	Bajka 2023	0	0	N/A

### Risk of bias assessment of included articles

The risk of bias assessment summary is depicted in Supplemental Fig. 1. Most studies (22 out of 28) had some concerns due to insufficient information on concealed allocation (Domain 1: Randomization process) and the absence of a pre-specified analysis plan (Domain 5: Selection of the reported results). Four studies had a high risk of bias due to missing outcome data (Domain 3). Only two studies had a low risk of bias across all domains.

### Qualitative synthesis

Articles that did not assess iAUC over 120 min or did not have comparable avCHO between the chickpea and comparator groups were excluded from the meta-analysis. Instead, a qualitative summary of glucose and insulin outcomes (iAUC and C_max_) is provided in Tables 4, 5, 6 and 7.

**Table 4:** Summary of acute chickpea consumption vs. comparator on postprandial glucose AUC

Author, Year	AUC Timing	Comparator Group		Chickpea Group		Chickpea vs. Comparator*	
		Description	Mean $\:\pm\:$ SD (mmol*min/L)	Description	Mean $\:\pm\:$ SD (mmol*min/L)	Reported *p*-value	Direction of Chickpeas
Chen 2022	Over 150 min	Brown rice (long chewing time)	269.7 $\:\pm\:$ 121.36	Boiled chickpeas (long chewing time)	122.9 $\:\pm\:$ 54.54	*p* < 0.05	↓
		Brown rice (short chewing time)	290.4 $\:\pm\:$ 111.31	Boiled chickpeas (short chewing time)	111.3 $\:\pm\:$ 60.33	*p* < 0.05	↓
Dandachy 2018	Over 210 min	Regular mankoushe	120.8 $\:\pm\:$ 88.74	Chickpeas mankoushe	119.3 $\:\pm\:$ 97.68	**NR**	↔
Johnston 2021	Over 120 min	Extruded corn snack	142 $\:\pm\:$ 89.74	Extruded corn snack with chickpea flour	110 $\:\pm\:$ 78.52	*p* < 0.05	↓
Khawaja 2012	Over 300 min	White bread (Healthy)	145.3 $\:\pm\:$ 332.09	Chickpea flour chapatti (Healthy)	122.4 $\:\pm\:$ 166.07	*p* = 0.002	↓
		White bread (T2DM)	868.2 $\:\pm\:$ 332.09	Chickpea flour chapatti (T2DM)	448.9 $\:\pm\:$ 166.06	*p* = 0.002	↓
Mehio 1997	Over 120 min	White bread	47.2 $\:\pm\:$ 30.27	Chickpea salad dip	6.1 $\:\pm\:$ 13.02	**NR**	**NR**
Mollard 2011	Over 260 min	Macaroni and cheese	298.1 $\:\pm\:$ 89.00	Canned chickpeas + pasta + tomato sauce	232 $\:\pm\:$ 113.50	*p* = 0.07	↔
Mollard 2012	Over 260 min	Macaroni pasta + tomato sauce	340.8 $\:\pm\:$ 174.89	Chickpeas + pasta + tomato sauce	220 $\:\pm\:$ 134.72	*p* < 0.05	↓
Mollard 2014	Over 135 min	White bread	239.7 $\:\pm\:$ 77.85	Canned chickpeas + tomato sauce	126.7 $\:\pm\:$ 65.84	*p* < 0.0001	↓
Panlasigui 1995	Over 60 min	White bread	94.7 $\:\pm\:$ 25.87	Boiled chickpeas	12.7 $\:\pm\:$ 8.62	*p* ≤ 0.01	↓
Voyatoglou 1995^	Over 210 min	White bread	1900.3 $\:\pm\:$ 331.03	Boiled chickpeas	1669.2 $\:\pm\:$ 215.42	*p* < 0.001	↓
Wong 2009	Over 120 min	White bread + tomato sauce	175.4 $\:\pm\:$ 49.57	Canned chickpeas + tomato sauce	111.7 $\:\pm\:$ 49.19	*p* < 0.001	↓
Zafar 2015	Over 90 min	Whole wheat bread	71.4 $\:\pm\:$ 24.11	Whole wheat bread (25% chickpea flour)	66.1 $\:\pm\:$ 21.49	**NR**	↔
				Whole wheat bread (35% chickpea flour)	39 $\:\pm\:$ 17.93	*p* < 0.05	↓
Zafar 2020	Over 90 min	White bread	116.3 $\:\pm\:$ 32.18	White bread (20% chickpea flour)	100.3 $\:\pm\:$ 32.83	*p* ≥ 0.05	↔
				White bread (30% chickpea flour)	77 $\:\pm\:$ 33.57	*p* < 0.05	↓
				White bread (40% chickpea flour)	74.7 $\:\pm\:$ 34.00	*p* < 0.05	↓

* Results are based on statistically significant differences between the chickpea and control group (*p* < 0.05), as reported by the authors in the original manuscript. ↑: increase; ↓: decrease; ↔: no change; NR: not reported

^ The unit for glucose area under the curve (AUC) reported by authors is mM.min

Abbreviations: SD, standard deviation; T2DM, Type 2 Diabetes Mellitus

**Table 5:** Summary of acute chickpea consumption vs. comparator on postprandial glucose C_max_

Author, Year	Comparator Group			Chickpea Group			Chickpea vs. Comparator*
	C_max_ Timing	Description	Mean $\:\pm\:$ SD (mmol/L)	C_max_ Timing	Description	Mean $\:\pm\:$ SD (mmol/L)	
Chen 2022	~ 83 min	Brown rice (long chewing time)	8.7 $\:\pm\:$ 1.73	~ 149 min	Boiled chickpeas (long chewing time)	6.2 $\:\pm\:$ 0.72	↓
	~ 72 min	Brown rice (short chewing time)	8.4 $\:\pm\:$ 1.68	~ 146 min	Boiled chickpeas (short chewing time)	6.2 $\:\pm\:$ 0.78	↓
Zafar 2015^	~ 30 min	Whole wheat bread	6.5 $\:\pm\:$ N/A	~ 30 min	Whole wheat bread (25% chickpea flour)	6.5 $\:\pm\:$ N/A	↔
					Whole wheat bread (35% chickpea flour)	6.1 $\:\pm\:$ 0.51	↔

* Results are based on statistically significant differences between the chickpea and control group (*p* < 0.05), as reported by the authors in the original manuscript. ↑: increase; ↓: decrease; ↔: no change

^ SD could not be extracted from figures due to overlap

Abbreviations: C_max_, maximum concentration; SD, standard deviation

**Table 6:** Summary of acute chickpea consumption vs. comparator on postprandial insulin AUC

Author, Year	AUC Timing	Comparator Group		Chickpea Group		Chickpea vs. Comparator*
		Description	Mean $\:\pm\:$ SD (pmol*min/L)	Description	Mean $\:\pm\:$ SD (pmol*min/L)	
Dandachy 2018	Over 210 min	Regular mankoushe	20960.0 $\:\pm\:$ 10799.48	Chickpeas mankoushe	16355.5 $\:\pm\:$ 7188.08	↔
Johnston 2021	Over 120 min	Extruded corn snack	15667.9 $\:\pm\:$ 8888.59	Extruded corn snack with chickpea flour	13556.6 $\:\pm\:$ 7932.44	↔
Mehio 1997	Over 120 min	White bread	22117.0 $\:\pm\:$ 14454.85	Chickpea salad dip	11902.3 $\:\pm\:$ 6173.33	**NR**
Voyatoglou 1995^	Over 210 min	White bread	75515.4 $\:\pm\:$ 19551.34	Boiled chickpeas	42156.0 $\:\pm\:$ 13063.44	↓

* Results are based on statistically significant differences between the chickpea and control group (*p* < 0.05), as reported by the authors in the original manuscript. ↑: increase; ↓: decrease; ↔: no change; NR: not reported

^ The unit for glucose AUC reported by authors is pM.min

Abbreviations: AUC, area under the curve; SD, standard deviation

**Table 7:** Summary of acute chickpea consumption vs. comparator on postprandial insulin C_max_

Author, Year	Comparator Group			Chickpea Group			Chickpea vs. Comparator*
	C_max_ Timing	Description	Geometric Mean $\:\pm\:$ GM SD Factor (pmol/L)	C_max_ Timing	Description	Geometric Mean $\:\pm\:$ GM SD Factor (pmol/L)	
Bajka 2023	~ 30 min	100% white flour bread + 20 g of no-added sugar strawberry jam	503.5 $\:\pm\:$ 11.74	~ 30 min	Bread containing 30% w/w cellular chickpea powder (CCP) + 20 g of no-added sugar strawberry jam	478.6 $\:\pm\:$ 11.67	↔
					Bread containing 60% w/w cellular chickpea powder (CCP) + 20 g of no-added sugar strawberry jam	360.9 $\:\pm\:$ 11.67	↓

* Results are based on statistically significant differences between the chickpea and control group (*p* < 0.05), as reported by the authors in the original manuscript. ↑: increase; ↓: decrease; ↔: no change

Abbreviation: CCP, cellular chickpea powder; C_max_, maximum concentration; GM, geometric mean; SD: standard deviation

#### Qualitative synthesis of glucose iAUC

Thirteen articles with 18 comparisons evaluated glucose iAUC [9, 10, 12, 38–47]. A majority of evidence suggests chickpeas mitigate postprandial glucose iAUC. Specifically, 72% (13/18) indicated that acute chickpea consumption lowers postprandial glucose iAUC compared to controls [9, 10, 12, 38, 39, 42, 44–47] (Table 4). Four comparisons (22%) reported no differences [40, 43, 46, 47], and one study did not report statistical differences [48].

#### Qualitative synthesis of glucose peak

Alternatively, findings were inconclusive for glucose C_max_. Two articles with four comparisons examined glucose C_max_ [38, 46] (Table 5). One study reported that chickpea consumption reduced peak glucose compared to controls [38]. The other study found no significant difference between whole wheat bread with 25% or 35% chickpea flour and control whole wheat bread [46].

#### Qualitative synthesis of insulin iAUC

Four articles with four comparisons assessed insulin iAUC, with inconsistent results [9, 40, 41, 45] (Table 6). One study reported a significant reduction in insulin AUC with chickpea consumption compared to white bread [45]. Two studies reported no change [9, 40], and one study did not report statistical differences [48].

#### Qualitative synthesis of insulin peak

One study with two comparisons assessed insulin C_max_ [49] (Table 7). Results were mixed: one comparison found no change (bread with 30% chickpea flour vs. white flour bread), while the other showed a reduction (bread with 60% chickpea flour vs. white flour bread) [49]. The evidence is insufficient to draw firm conclusions.

### Primary meta-analysis

#### Effect of chickpeas on glucose iAUC

**Fig. 2:**
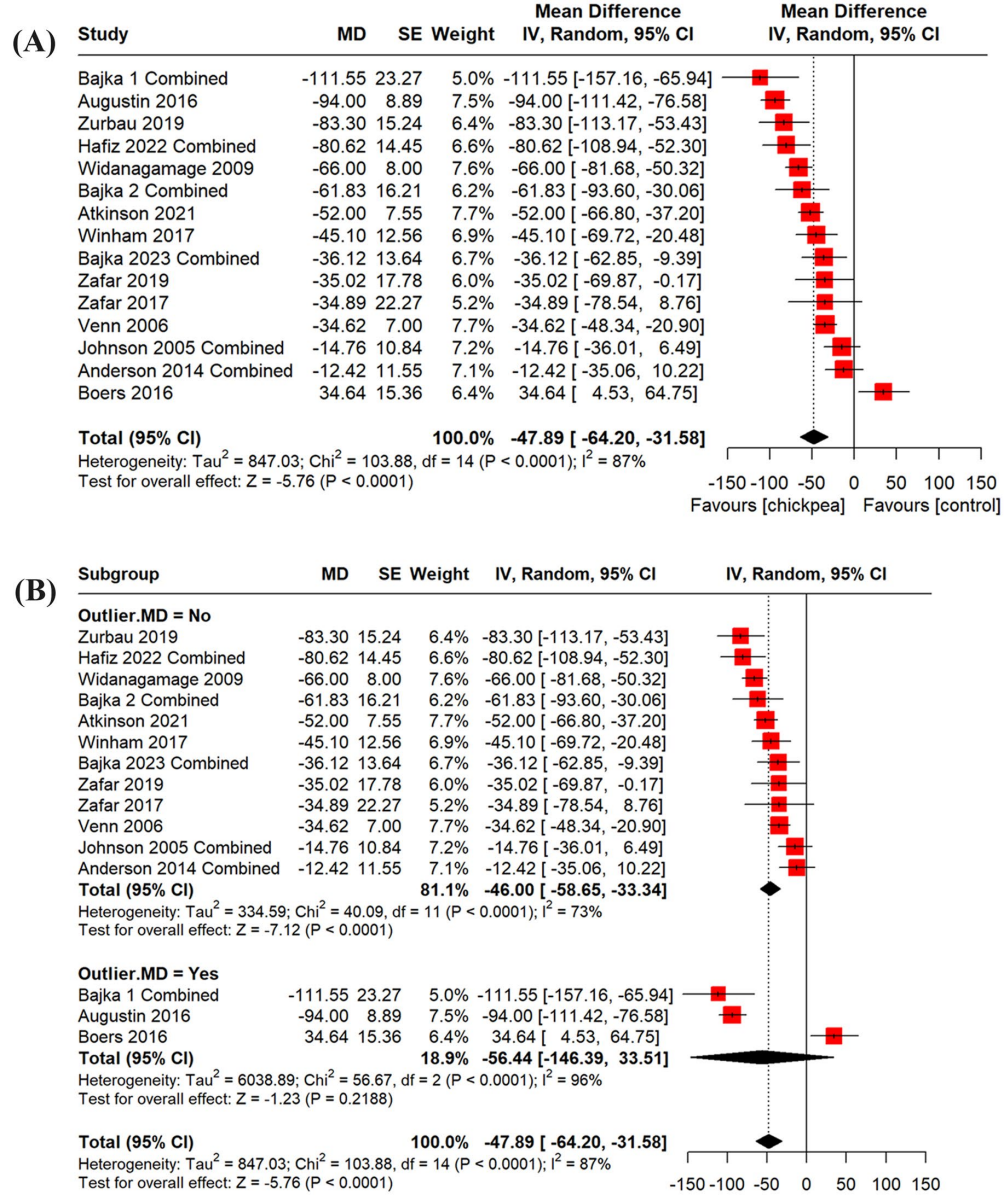
Pooled effect estimates of the effect of chickpea on the incremental area under the curve (iAUC) for blood glucose. Pooled effect estimates are expressed as mean difference with 95% CI with (**A**) or without (**B**) outliers. Pooled analyses were conducted using the generic inverse variance method with random effects models. Inter-study heterogeneity was assessed using the Cochran Q statistic and quantified using the I^2^ statistic, where I^2^ ≥ 50% and *P* < 0.10 were considered evidence of substantial heterogeneity

A total of 15 studies were included in the quantitative analysis of glucose iAUC [6, 11, 37, 49–59]. The studies covered UK (*n* = 5), North America (*n* = 4), Australasia (*n* = 3), Kuwait (*n* = 2), and Sri Lanka (*n* = 1). All studies were in adults whereby three were exclusively in females, one was exclusively in males, and the remaining were in both males and females. Interventions ranged from whole, cooked chickpeas (*n* = 9), beverage made from chickpea powder (*n* = 3), bread made with chickpea flour (*n* = 2), or hummus (*n* = 1). Of the 15 studies included, two had low risk of bias, three had high risk of bias and the remaining had some concerns (Supplemental Fig. 1). Studies with high risk of bias had potential limitations related to > 20% early termination. Chickpea ingestion significantly lowered glucose iAUC compared to controls (MD: -47.89 [95% CI: -64.20, -31.58], *p* < 0.0001) with substantial heterogeneity (I² = 87%, *p* < 0.0001) (Fig. 2A). Sensitivity analysis identified three outliers (Fig. 2B). Removing these reduced heterogeneity (I² = 73%, *p* < 0.0001) but did not alter the direction or significance of the effect (MD: -46.00 [95% CI: -58.65, -33.34], *p* < 0.0001).

#### Effect of chickpeas on glucose C_max_

**Fig. 3:**
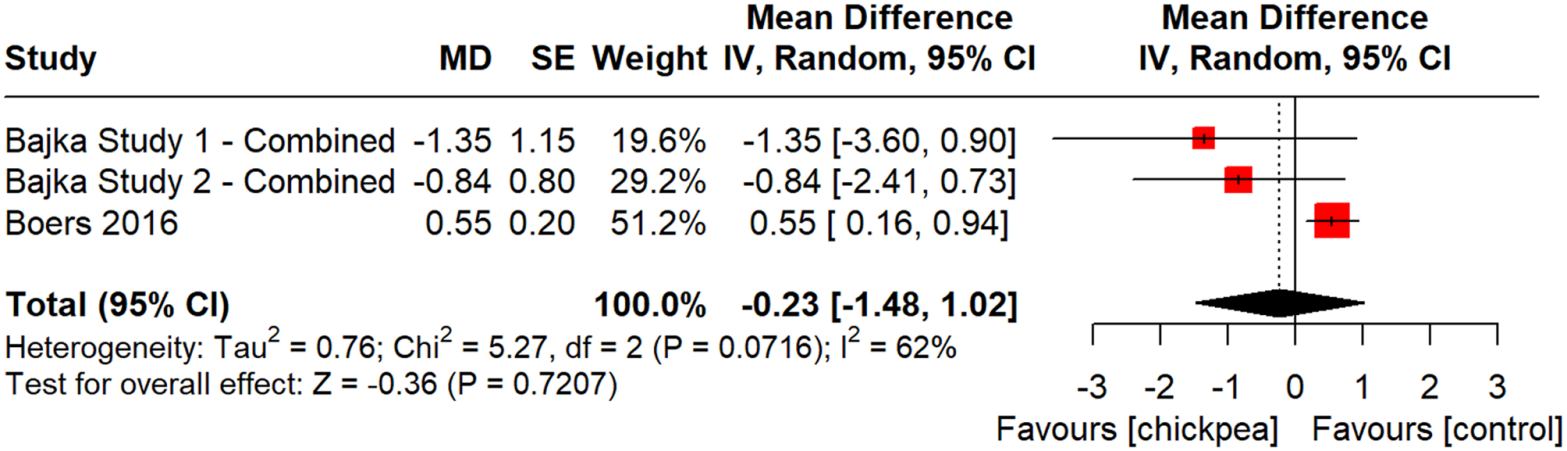
Pooled effect estimates of the effect of chickpea on the maximum concentration (C_max_) for blood glucose. Pooled effect estimates are expressed as mean difference with 95% CI. Pooled analyses were conducted using the generic inverse variance method with random effects models. Inter-study heterogeneity was assessed using the Cochran Q statistic and quantified using the I^2^ statistic, where I^2^ ≥ 50% and *P* < 0.10 were considered evidence of substantial heterogeneity

A total of five studies reported on glucose C_max_. However, one study was excluded because it presented the geometric mean and geometric standard deviation rather than the mean and standard deviation [49] and another was excluded due to not reporting any identifiable/extractable SD nor SEM [53]. The remaining three studies were all conducted in the UK in male and female adults [37, 52]. One provided chickpea powder in the form of a beverage, another provided bread made from chickpea powder, and the third provided bread made from chickpea flour. One study had low risk of bias, one had high risk of bias and one had some concerns (Supplemental Fig. 1). The study with high risk of bias had potential limitations related to > 20% early termination. Chickpea ingestion did not significantly affect glucose C_max_ (MD: -0.23 [95% CI: -1.48, 1.02], *p* = 0.7207) with substantial heterogeneity (I² = 62%, *p* = 0.0716) (Fig. 3).

#### Effect of chickpeas on insulin iAUC

**Fig. 4:**
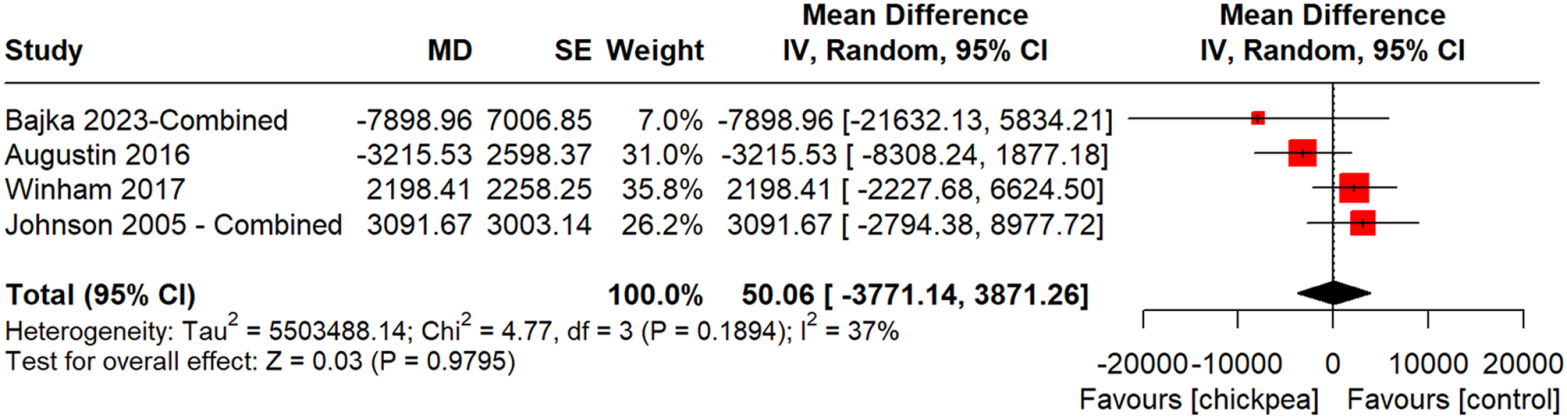
Pooled effect estimates of the effect of chickpea on the incremental area under the curve (iAUC) for blood insulin. Pooled effect estimates are expressed as mean difference with 95% CI. Pooled analyses were conducted using the generic inverse variance method with random effects models. Inter-study heterogeneity was assessed using the Cochran Q statistic and quantified using the I^2^ statistic, where I^2^ ≥ 50% and *P* < 0.10 were considered evidence of substantial heterogeneity

Of the four studies analyzed, two were conducted in North America, one in the UK, and another in Australia [49, 51, 54, 56]. One was exclusively in female adults and the remaining three were in both male and female adults. Interventions included bread containing chickpea powder or flour (*n* = 2), whole cooked chickpeas (*n* = 1) or hummus (*n* = 1). One study was rated as having low risk of bias, another as having high risk of bias, and the remaining two had some concerns (Supplemental Fig. 1). The study with high risk of bias had potential limitations related to > 20% early termination. Chickpea ingestion did not significantly alter insulin iAUC compared to controls (MD: 50.06 [95% CI: -3771.14, 3871.26], *p* = 0.9795), with moderate heterogeneity (I² = 37%, *p* = 0.1894) (Fig. 4). There were insufficient studies to perform a meta-analysis for insulin C_max_.

### Publication bias analyses

The funnel plot for glucose iAUC showed no evidence of publication bias (Supplemental Fig. 2) and Egger’s test was also non-significant for glucose iAUC (intercept = 0.18, *p* = 0.8570). Due to the small number of studies (*n* = 3), publication bias was not evaluated for glucose C_max_ or insulin iAUC.

### GRADE assessment

A GRADE for each outcome is shown in Supplemental Table 2. Our certainty in the evidence was very low for postprandial glucose C_max_, and low quality for both postprandial glucose and insulin iAUC. Quality of evidence was primarily hindered by substantial and significant heterogeneity, as well as small study size, which influenced imprecision and ability to evaluate publication bias. Most studies were judged as having some risk of bias concerns, mainly due to possible selective reporting. However, no downgrades were applied because these concerns were primarily based on failure to report if a statistical analysis plan was in place before the unblinding of data, which is unlikely to lower confidence in effect.

## Discussion

In this systematic review and meta-analysis, we evaluated the effects of acute chickpea consumption on postprandial glucose and insulin responses in controlled, crossover, single-meal feeding trials. Our meta-analyses found that chickpea consumption significantly reduced postprandial glucose iAUC compared to avCHO-matched controls in healthy adults without T2DM, albeit with low level of certainty. No significant effects were observed for glucose C_max_ or insulin iAUC; however, the number of studies for these later two outcomes were limited. These findings suggest that incorporating chickpeas into regular diets could help improve glycemic control, which is crucial for reducing the risk of T2DM. The results confirm that chickpeas have an intrinsic glucoregulatory effect, independent of differences in digestible carbohydrate content.

Our findings aligned with previous research investigating the effects of pulses on glycemic control [16, 21, 22]. A 2009 meta-analysis of 41 RCTs reported that pulses, either alone or incorporated into low-GI or high-fiber diets (2 weeks– 1 year), reduced fasting glucose, insulin, and HbA1c in adults with or without T2DM [22]. Similarly, a 2022 meta-analysis of 65 RCTs concluded that chronic pulse consumption (3–16 weeks) significantly reduced fasting glucose in normoglycemic adults and improved HbA1c and HOMA-IR in individuals with T2DM [21]. This meta-analysis also showed that pulses reduced peak postprandial glucose in individuals with and without T2DM, with chickpeas demonstrating the third-largest effect size for postprandial glucose reduction [21]. More recently, a small 2023 meta-analysis found that chickpeas specifically lowered postprandial glucose iAUC compared to wheat (*n* = 3 studies) and potatoes (*n* = 2 studies) [16]. Our study expands on these findings by including a larger number of studies (15 vs. 6) and controlling for avCHO, which the 2023 review did not address [16]. Consistent with the 2023 review [16], we observed lower postprandial glucose iAUC with chickpea without any changes in postprandial insulin iAUC.

In concept, the reduction in postprandial glucose iAUC independent of avCHO intake observed with chickpea consumption, can be mechanistically explained by the soluble and insoluble fiber content, as well as bioactive compounds like polyphenols, saponins, and phytosterols, naturally present in chickpeas. Soluble fiber forms a viscous gel in the digestive tract, delaying gastric emptying and glucose absorption [60]. Bioactive compounds may also modulate digestion and absorption. For instance, polyphenols may inhibit digestive enzymes like α-amylase and α-glucosidase, slowing carbohydrate breakdown and absorption. Saponins and phytosterols may modulate glucose transporters and insulin signaling pathways, contributing to improved glycemic control [61–63]. Insoluble fiber can inhibit excessive glucose adsorption and decrease starch hydrolysis, influencing glycemic control [64]. Additionally, insoluble fiber and resistant starches resist digestion in the small intestine and reach the colon, where they are fermented by gut microbiota. This fermentation produces short-chain fatty acids (SCFAs), which enhance insulin sensitivity, reduce hepatic glucose production, and stimulate the release of incretin hormones like glucagon-like peptide-1 (GLP-1) [65]. These digestive and metabolic properties of chickpea nutrients would suggest a reduction in glucose C_max_ and insulin iAUC, which were insignificant findings in our current analysis. This misalignment may be due to low power from limited sample size. Additional studies are needed to better elucidate the mechanisms of glycemic control by chickpea.

Given the glucoregulatory effects observed, consuming chickpeas with or in place of high-GI staples like white bread or rice may be a simple dietary approach for individuals seeking to manage their blood glucose levels. The availability of various chickpea products allows for easy incorporation of chickpeas into the habitual diet; however, processing methods such as boiling, milling to produce chickpea flour, or pureeing (as in hummus) may alter the fiber structure and nutrient availability and thus, its glucoregulatory properties. Future research should compare different processing methods to identify which forms of chickpeas offer the greatest glycemic benefits.

A major strength of this systematic review and meta-analysis is the use of a PICOS framework that balanced standardization with flexibility. By including only crossover RCTs, using avCHO-matched comparators, and standardizing iAUC to 120 min, we minimized confounding variables and enhanced the robustness of our findings. These elements allowed us to isolate the glucoregulatory effects of chickpeas from differences in carbohydrate content, increasing the translational potential of our results. However, our study has limitations. While we controlled for avCHO, we could not speculate on the influence of other nutritive components, such as dietary fat or protein, which are known to affect postprandial glucose responses [66]. Future research should explore chickpea consumption in mixed-meal settings to understand how macronutrient interactions influence glycemic responses in real-world scenarios. Additionally, although we used a generic population parameter, the final set of included studies only involved healthy adults, limiting the generalizability of our findings to individuals with T2DM or other metabolic disorders. Future studies should include metabolically unhealthy populations to assess whether the observed effects extend to these groups. We also observed substantial heterogeneity in glucose iAUC and glucose C_max_ outcomes, which may be due to differences in BMI, age, or the form of chickpea interventions (effect of processing methods). Although we intended to investigate the effects of these study variations, the small number of studies in each subgroup did not allow for meaningful conclusions. For processing, lessons from other foods, such as oats, suggest that whole food forms like boiled chickpeas might maintain lower GI values and more favorable glucoregulatory effects compared to products like hummus or chickpea flour that have undergone greater structural modification [67]. Future studies should explore how processing affects the glycemic properties of chickpeas to guide dietary recommendations. There is also the potential to use the GI as a metric to address heterogeneity stemming from processing methods by providing a standardized approach to evaluate the blood glucose-raising potential of these carbohydrate-rich foods. Lastly, the included studies only evaluated acute, single-meal effects of chickpeas, limiting our ability to comment on their long-term impact on glucose regulation. Long-term intervention trials are warranted to determine if regular chickpea consumption can sustainably improve glycemic control.

In summary, this meta-analysis found that chickpea consumption reduces postprandial glucose iAUC independent of differences in digestible carbohydrate content. This finding is conceptually supported by the unique properties of chickpeas, including their low glycemic index, fiber content, bioactive compounds, and prebiotic effects, though more research is needed to delineate the potency of chickpea consumption across multiple metrics of glycemic control. Encouraging the inclusion of chickpeas in everyday diets may represent a simple, accessible strategy for managing postprandial glycemia, ultimately contributing to the prevention of T2DM and other related conditions.

## Electronic supplementary material

Below is the link to the electronic supplementary material.

Supplementary Material 1

## Data Availability

The datasets used and/or analyzed during the current study are available from the corresponding author on reasonable request.

## Abbreviations

avCHO: available carbohydrate
CCP: cellular chickpea powder
CGM: continuous glucose monitoring
C_max_: maximum concentration
CVD: cardiovascular disease
GI: glycemic index
iAUC: incremental Area Under the Curve
ICP: intact cell powder
MD: mean difference
n/a: not available
NR: not reported
RCP: ruptured cell powder
SMD: standard mean difference
T2DM: Type 2 Diabetes Mellitus
UK: United Kingdom
USA: United States of America

## References

[1] Diabetes Mellitus. A major risk factor for cardiovascular disease. Circ. 1999;100(10):1132–3. 10.1161/01.cir.100.10.1132 10477541

[2] Center for Disease Control, National Center for Health Statistics. Leading Causes of Death 2024 [updated 2024. Available from: https://www.cdc.gov/nchs/fastats/leading-causes-of-death.htm

[3] World Health Organization. The top 10 causes of death 2024 [updated 7 August 2024. Available from: https://www.who.int/news-room/fact-sheets/detail/the-top-10-causes-of-death

[4] Clar C, Al-Khudairy L, Loveman E, Kelly SA, Hartley L, Flowers N, et al. Low glycaemic index diets for the prevention of cardiovascular disease. Cochrane Database Syst Rev. 2017;7:CD004467. 28759107 10.1002/14651858.CD004467.pub3 PMC6483287

[5] Livesey G, Taylor R, Hulshof T, Howlett J. Glycemic response and health–a systematic review and meta-analysis: relations between dietary glycemic properties and health outcomes. Am J Clin Nutr. 2008;87(1):S258–68. 10.1093/ajcn/87.1.258S 18175766

[6] Zafar MI, Mills KE, Zheng J, Regmi A, Hu SQ, Gou L, et al. Low-glycemic index diets as an intervention for diabetes: a systematic review and meta-analysis. Am J Clin Nutr. 2019;110(4):891–902. 31374573 10.1093/ajcn/nqz149

[7] Rajagukguk YV, Arnold M, Gramza-Michałowska A. Pulse probiotic superfood as Iron status improvement agent in active Women-A review. Molecules. 2021;26(8). 10.3390/molecules26082121 PMC8067853 33917113

[8] Becerra-Tomás N, Díaz-López A, Rosique-Esteban N, Ros E, Buil-Cosiales P, Corella D, et al. Legume consumption is inversely associated with type 2 diabetes incidence in adults: A prospective assessment from the PREDIMED study. Clinical nutrition (Edinburgh. Scotland). 2018;37(3):906–13. 10.1016/j.clnu.2017.03.015 28392166

[9] Johnston AJ, Mollard RC, Dandeneau D, MacKay DS, Ames N, Curran J, et al. Acute effects of extruded pulse snacks on glycemic response, insulin, appetite, and food intake in healthy young adults in a double blind, randomized, crossover trial. Applied physiology, nutrition, and metabolism = physiologie appliquee. Nutr Et Metab. 2021;46(7):704–10. 10.1139/apnm-2020-0572 33347383

[10] Mollard RC, Wong CL, Luhovyy BL, Cho F, Anderson GH. Second-meal effects of pulses on blood glucose and subjective appetite following a standardized meal 2 h later. Applied physiology, nutrition, and metabolism = physiologie appliquee. Nutr Et Metab. 2014;39(7):849–51. 10.1139/apnm-2013-0523 24797207

[11] Widanagamage RD, Ekanayake S, Welihinda J. Carbohydrate-rich foods: glycaemic indices and the effect of constituent macronutrients. Int J Food Sci Nutr. 2009;60(Suppl 4):215–23. 19418327 10.1080/09637480902849195

[12] Wong CL, Mollard RC, Zafar TA, Luhovyy BL, Anderson GH. Food intake and satiety following a serving of pulses in young men: effect of processing, recipe, and pulse variety. J Am Coll Nutr. 2009;28(5):543–52. 20439550 10.1080/07315724.2009.10719786

[13] American Diabetes Association, Bantle JP, Wylie-Rosett J, Albright AL, Apovian CM, Clark NG, et al. Nutrition recommendations and interventions for diabetes: a position statement of the American diabetes association. Diabetes Care. 2008;31(Suppl 1):S61–78. 18165339 10.2337/dc08-S061

[14] Bhattacharyya OK, Shah BR, Booth GL. Management of cardiovascular disease in patients with diabetes: the 2008 Canadian diabetes association guidelines. CMAJ. 2008;179(9):920–6. 18801878 10.1503/cmaj.080554 PMC2565732

[15] Mann JI, De Leeuw I, Hermansen K, Karamanos B, Karlstrom B, Katsilambros N, et al. Evidence-based nutritional approaches to the treatment and prevention of diabetes mellitus. Nutr Metab Cardiovasc Dis. 2004;14(6):373–94. 15853122 10.1016/s0939-4753(04)80028-0

[16] Nam T, Kim A, Oh Y. Effectiveness of Chickpeas on blood sugar: a systematic review and meta-analysis of randomized controlled trials. Nutrients. 2023;15(21):4556. 37960209 10.3390/nu15214556 PMC10647263

[17] Begum N, Khan QU, Liu LG, Li W, Liu D, Haq IU. Nutritional composition, health benefits and bio-active compounds of Chickpea (Cicer arietinum L). Front Nutr. 2023;10:1218468. 37854353 10.3389/fnut.2023.1218468 PMC10580981

[18] Singh B, Singh JP, Shevkani K, Singh N, Kaur A. Bioactive constituents in pulses and their health benefits. J Food Sci Technol. 2017;54:858–70. 28303037 10.1007/s13197-016-2391-9 PMC5336453

[19] Pearce M, Fanidi A, Bishop TRP, Sharp SJ, Imamura F, Dietrich S, et al. Associations of total legume, pulse, and soy consumption with incident type 2 diabetes: federated Meta-Analysis of 27 studies from diverse world regions. J Nutr. 2021;151(5):1231–40. 33693815 10.1093/jn/nxaa447 PMC8112771

[20] Viguiliouk E, Glenn AJ, Nishi SK, Chiavaroli L, Seider M, Khan T, et al. Associations between dietary pulses alone or with other legumes and cardiometabolic disease outcomes: an umbrella review and updated systematic review and Meta-analysis of prospective cohort studies. Adv Nutr. 2019;10(Suppl4):S308–19. 31728500 10.1093/advances/nmz113 PMC6855952

[21] Hafiz MS, Campbell MD, O’Mahoney LL, Holmes M, Orfila C, Boesch C. Pulse consumption improves indices of glycemic control in adults with and without type 2 diabetes: a systematic review and meta-analysis of acute and long-term randomized controlled trials. Eur J Nutr. 2022:1–16. 10.1007/s00394-021-02685-y PMC8854292 34585281

[22] Sievenpiper JL, Kendall CW, Esfahani A, Wong JM, Carleton AJ, Jiang HY, et al. Effect of non-oil-seed pulses on glycaemic control: a systematic review and meta-analysis of randomised controlled experimental trials in people with and without diabetes. Diabetologia. 2009;52(8):1479–95. 19526214 10.1007/s00125-009-1395-7

[23] Food and Drug Administration. Scientific evaluation of the evidence on the beneficial physiological effects of isolated or synthetic Non-Digestible carbohydrates submitted as a citizen petition (21 CFR 10.30): guidance for industry. In: U.S. Department of Health and Human Services, editor.; 2018.

[24] Higgins J, Thomas J, Chandler J, Cumpston M, Li T, Page M et al. Cochrane Handbook for Systematic Reviews of Interventions version 6.3 (updated February 2022) 2022 [Available from: www.training.cochrane.org/handbook

[25] Page MJ, McKenzie JE, Bossuyt PM, Boutron I, Hoffmann TC, Mulrow CD, et al. The PRISMA 2020 statement: an updated guideline for reporting systematic reviews. BMJ. 2021;372:n71. 33782057 10.1136/bmj.n71 PMC8005924

[26] Wolever TM. Effect of blood sampling schedule and method of calculating the area under the curve on validity and precision of glycaemic index values. Br J Nutr. 2004;91(2):295–300. 14756916 10.1079/bjn20031054

[27] Bielefeld D, Grafenauer S, Rangan A. The effects of legume consumption on markers of glycaemic control in individuals with and without diabetes mellitus: A systematic literature review of randomised controlled trials. Nutrients. 2020;12(7). 10.3390/nu12072123 PMC7400945 32708949

[28] Higgins J. The Cochrane collaboration’s tool for assessing risk of Bias in randomised trials. Cochrane Collab. 2011(343):d5928. 10.1136/bmj.d5928 PMC3196245 22008217

[29] Boon MH, Thomson H. The effect direction plot revisited: application of the 2019 Cochrane handbook guidance on alternative synthesis methods. Res Synth Methods. 2021;12(1):29–33. 32979023 10.1002/jrsm.1458 PMC7821279

[30] Team RC. R: A Language and environment for statistical computing. Vienna, Austria: Foundation for Statistical Computing; 2013.

[31] Harrer M, Cuijpers P, Furukawa TA, Ebert DD, Chapter. Between-Study heterogeneity. Doing meta-analysis with R: A hands-on guide. Volume 5. Boca Raton, FL and London: Chapman and Hall/CRC; 2021.

[32] Egger M, Davey Smith G, Schneider M, Minder C. Bias in meta-analysis detected by a simple, graphical test. BMJ. 1997;315(7109):629–34. 9310563 10.1136/bmj.315.7109.629 PMC2127453

[33] Begg CB, Mazumdar M. Operating characteristics of a rank correlation test for publication bias. Biometrics. 1994;50(4):1088–101. 7786990

[34] Zwetsloot PP, Van Der Naald M, Sena ES, Howells DW, IntHout J, De Groot JA et al. Standardized mean differences cause funnel plot distortion in publication bias assessments. Elife. 2017;6. 10.7554/eLife.24260 PMC5621838 28884685

[35] Harrer M, Cuijpers P, Furukawa TA, Ebert DD. Chapter 9: publication bias. Doing Meta-Analysis with R: A Hands-On guide. Boca Raton, FL and London: Chapman & Hall/CRC; 2021.

[36] Pustejovsky JE, Rodgers MA. Testing for funnel plot asymmetry of standardized mean differences. Res Synth Methods. 2019;10(1):57–71. 30506832 10.1002/jrsm.1332

[37] Boers HM, MacAulay K, Murray P, Seijen Ten Hoorn J, Hoogenraad AR, Peters HPF, et al. Efficacy of different fibres and flour mixes in South-Asian flatbreads for reducing post-prandial glucose responses in healthy adults. Eur J Nutr. 2017;56(6):2049–60. 27324141 10.1007/s00394-016-1242-9 PMC5579182

[38] Chen Y, Stieger M, Capuano E, Forde CG, van der Haar S, Ummels M, et al. Influence of oral processing behaviour and bolus properties of brown rice and Chickpeas on in vitro starch digestion and postprandial glycaemic response. Eur J Nutr. 2022;61(8):3961–74. 35773354 10.1007/s00394-022-02935-7 PMC9596526

[39] Khawaja KI, Fatima A, Mian SA, Mumtaz U, Moazzum A, Ghias M, et al. Glycaemic, insulin and Ghrelin responses to traditional South Asian flatbreads in diabetic and healthy subjects. Br J Nutr. 2012;108(10):1810–7. 22243983 10.1017/S0007114511007264

[40] Dandachy S, Mawlawi H, Chedid M, El-Mallah C, Obeid O. Impact of pre-processed Chickpea flour incorporation into Mankoushe on appetite hormones and scores. Foods. 2018;7(10):173. 30347703 10.3390/foods7100173 PMC6209887

[41] Mehio Z, Baba NH, Habbal Z. Glycemic and insulinemic responses of normal subjects to selected meals commonly consumed in the middle East. J Nutr Environ Med. 1997;7(4):275–86.

[42] Mollard R, Zykus A, Luhovyy B, Nunez M, Wong C, Anderson G. The acute effects of a pulse-containing meal on glycaemic responses and measures of satiety and satiation within and at a later meal. Br J Nutr. 2012;108(3):509–17. 22054112 10.1017/S0007114511005836

[43] Mollard RC, Wong CL, Luhovyy BL, Anderson GH. First and second meal effects of pulses on blood glucose, appetite, and food intake at a later meal. Applied physiology, nutrition, and metabolism = physiologie appliquee. Nutr Et Metab. 2011;36(5):634–42. 10.1139/h11-071 21957874

[44] Panlasigui LN, Panlilio LM, Madrid JC. Glycaemic response in normal subjects to five different legumes commonly used in the Philippines. Int J Food Sci Nutr. 1995;46(2):155–60. 7621088 10.3109/09637489509012544

[45] Voyatzoglou D, Loupa C, Philippides P, Siskoudis P, Kitsou E, Alevizou V, et al. Insulin response to legumes in type 2 diabetic persons. Eur J Intern Med. 1995;6:201–3.

[46] Zafar TA, Al-Hassawi F, Al-Khulaifi F, Al-Rayyes G, Waslien C, Huffman FG. Organoleptic and glycemic properties of chickpea-wheat composite breads. Int J Food Sci. 2015;52:2256–63. 10.1007/s13197-013-1192-7 PMC4375205 25829607

[47] Zafar TA, Aldughpassi A, Al-Mussallam A, Al-Othman A. Microstructure of whole wheat versus white flour and wheat-Chickpea flour blends and dough: impact on the glycemic response of Pan bread. Int J Food Sci. 2020;2020(1):8834960. 33083447 10.1155/2020/8834960 PMC7557900

[48] Zeina MNHBZH. Glycemic and insulinemic responses of normal subjects to selected meals commonly consumed in the middle East. J Nutr Med. 1997;7(4):275–86.

[49] Bajka BH, Pinto AM, Perez-Moral N, Saha S, Ryden P, Ahn-Jarvis J, et al. Enhanced secretion of satiety-promoting gut hormones in healthy humans after consumption of white bread enriched with cellular Chickpea flour: A randomized crossover study. Am J Clin Nutr. 2023;117(3):477–89. 36811474 10.1016/j.ajcnut.2022.12.008 PMC10131617

[50] Anderson GH, Liu Y, Smith CE, Liu TT, Nunez MF, Mollard RC, et al. The acute effect of commercially available pulse powders on postprandial glycaemic response in healthy young men. Br J Nutr. 2014;112(12):1966–73. 25327223 10.1017/S0007114514003031

[51] Augustin LS, Chiavaroli L, Campbell J, Ezatagha A, Jenkins AL, Esfahani A, et al. Post-prandial glucose and insulin responses of hummus alone or combined with a carbohydrate food: a dose-response study. Nutr J. 2016;15:13. 26818604 10.1186/s12937-016-0129-1 PMC4730744

[52] Bajka BH, Pinto AM, Ahn-Jarvis J, Ryden P, Perez-Moral N, van der Schoot A, et al. The impact of replacing wheat flour with cellular legume powder on starch bioaccessibility, glycaemic response and bread roll quality: A double-blind randomised controlled trial in healthy participants. Food Hydrocoll. 2021;114:106565. 33941996 10.1016/j.foodhyd.2020.106565 PMC7859705

[53] Hafiz MS, Campbell MD, Orsi NM, Mappa G, Orfila C, Boesch C. Impact of food processing on postprandial glycaemic and appetite responses in healthy adults: a randomized, controlled trial. Food Funct. 2022;13(3):1280–90. 35024710 10.1039/d1fo02304g

[54] Johnson SK, Thomas SJ, Hall RS. Palatability and glucose, insulin and satiety responses of Chickpea flour and extruded Chickpea flour bread eaten as part of a breakfast. Eur J Clin Nutr. 2005;59(2):169–76. 15483639 10.1038/sj.ejcn.1602054

[55] Venn BJ, Wallace AJ, Monro JA, Perry T, Brown R, Frampton C, et al. The glycemic load estimated from the glycemic index does not differ greatly from that measured using a standard curve in healthy volunteers. J Nutr. 2006;136(5):1377–81. 16614433 10.1093/jn/136.5.1377

[56] Winham DM, Hutchins AM, Thompson SV. Glycemic response to black beans and Chickpeas as part of a rice meal: A randomized Cross-Over trial. Nutrients. 2017;9(10). 10.3390/nu9101095 PMC5691712 28976933

[57] Zafar TA, Kabir Y. Chickpeas suppress postprandial blood glucose concentration, and appetite and reduce energy intake at the next meal. J Food Sci Technol. 2017;54(4):987–94. 28303049 10.1007/s13197-016-2422-6 PMC5336455

[58] Zurbau A, Jenkins AL, Jovanovski E, Au-Yeung F, Bateman EA, Brissette C, et al. Acute effect of equicaloric meals varying in glycemic index and glycemic load on arterial stiffness and glycemia in healthy adults: a randomized crossover trial. Eur J Clin Nutr. 2019;73(1):79–85. 29777241 10.1038/s41430-018-0182-2

[59] Atkinson FS, Khan JH, Brand-Miller JC, Eberhard J. The impact of carbohydrate quality on dental plaque pH: does the glycemic index of starchy foods matter for dental health?? Nutrients. 2021;13(8). 10.3390/nu13082711 PMC8401118 34444871

[60] Giuntini EB, Sardá FAH, de Menezes EW. The effects of soluble dietary fibers on glycemic response: an overview and futures perspectives. Foods. 2022;11(23):3934. 36496742 10.3390/foods11233934 PMC9736284

[61] Kim Y, Keogh JB, Clifton PM. Polyphenols and glycemic control. Nutrients. 2016;8(1):17. 26742071 10.3390/nu8010017 PMC4728631

[62] Prasad M, Jayaraman S, Eladl MA, El-Sherbiny M, Abdelrahman MAE, Veeraraghavan VP, et al. A comprehensive review on therapeutic perspectives of phytosterols in insulin resistance: a mechanistic approach. Molecules. 2022;27(5):1595. 35268696 10.3390/molecules27051595 PMC8911698

[63] Zhou Y, Xu B. New insights into anti-diabetes effects and molecular mechanisms of dietary saponins. Crit Rev Food Sci Nutr. 2023;63(33):12372–97. 35866515 10.1080/10408398.2022.2101425

[64] Zhang G, Wang D, Ding Y, Zhang J, Ding Y, Lyu F. Effect and mechanism of insoluble dietary fiber on postprandial blood sugar regulation. Trends Food Sci Technol. 2024:104354.

[65] Portincasa P, Bonfrate L, Vacca M, De Angelis M, Farella I, Lanza E, et al. Gut microbiota and short chain fatty acids: implications in glucose homeostasis. Int J Mol Sci. 2022;23(3):1105. 35163038 10.3390/ijms23031105 PMC8835596

[66] Heer M, Egert S. Nutrients other than carbohydrates: their effects on glucose homeostasis in humans. Diabetes Metab Res Rev. 2015;31(1):14–35. 24510463 10.1002/dmrr.2533

[67] Tosh SM, Chu Y. Systematic review of the effect of processing of whole-grain oat cereals on glycaemic response. Br J Nutr. 2015;114(8):1256–62. 26330200 10.1017/S0007114515002895

