# Supplementary material for: Chickpea attenuates postprandial blood glucose responses: a systematic review and meta-analysis
Source: Nutr J. 2025 Jul 14;24:111. doi: 10.1186/s12937-025-01176-8 (PMC12261582; doi:10.1186/s12937-025-01176-8)
Supplement: Supplementary file 1 — Supplementary Material 1 [file 12937_2025_1176_MOESM1_ESM.docx]

**Supplemental Table 1.** Search terms and results for a systematically searched literature review

| **Source** | **Search Terms** | **Results** |
| --- | --- | --- |
| PubMed | (("chick"[All Fields] OR "chick s"[All Fields] OR "chicks"[All Fields]) AND ("pisum sativum"[MeSH Terms] OR ("pisum"[All Fields] AND "sativum"[All Fields]) OR "pisum sativum"[All Fields] OR "pea"[All Fields])) OR (("chick"[All Fields] OR "chick s"[All Fields] OR "chicks"[All Fields]) AND ("pisum sativum"[MeSH Terms] OR ("pisum"[All Fields] AND "sativum"[All Fields]) OR "pisum sativum"[All Fields] OR "pea"[All Fields])) OR ("chickpea s"[All Fields] OR "cicer"[MeSH Terms] OR "cicer"[All Fields] OR "chickpea"[All Fields] OR "chickpeas"[All Fields]) OR ("cicer"[MeSH Terms] OR "cicer"[All Fields] OR ("chick"[All Fields] AND "peas"[All Fields]) OR "chick peas"[All Fields]) OR ("cicer"[MeSH Terms] OR "cicer"[All Fields] OR ("cicer"[All Fields] AND "arietinum"[All Fields]) OR "cicer arietinum"[All Fields]) OR ("cicer"[MeSH Terms] OR "cicer"[All Fields] OR "garbanzo"[All Fields] OR (("cicer"[MeSH Terms] OR "cicer"[All Fields] OR "garbanzo"[All Fields]) AND "bean"[All Fields])) OR "falafel"[All Fields] OR "hummus"[All Fields] | **3,818** |
| Central | ((((chick pea or chick pea or chickpeas or chick peas) OR (cicer arietinum)) OR (garbanzo or garbanzo bean)) OR (falafel)) OR (hummus) | **192** |
| Embase | **('chick pea' OR (('chick'/exp OR chick) AND ('pea'/exp OR pea))) OR chickpeas OR ('chick pea' OR (('chick'/exp OR chick) AND ('pea'/exp OR pea))) OR ('chick peas' OR (('chick'/exp OR chick) AND ('peas'/exp OR peas))) OR ('cicer arietinum'/exp OR 'cicer arietinum' OR (('cicer'/exp OR cicer) AND arietinum)) OR ('garbanzo'/exp OR garbanzo) OR ('garbanzo bean'/exp OR 'garbanzo bean' OR (('garbanzo'/exp OR garbanzo) AND ('bean'/exp OR bean))) OR falafel OR hummus** | 3,548 |

Searches were conducted from inception to March 21, 2024

**Supplemental Table 2.** GRADE Summary of Findings

| Chickpeas compared to avCHO for postprandial glucose and insulin management | | | | | | | | | | | | | | | |
| --- | --- | --- | --- | --- | --- | --- | --- | --- | --- | --- | --- | --- | --- | --- | --- |
| Population | | | Non-pregnant and non-lactating humans of any health status or age | | | | | | | | | | | | |
| Intervention | | | Groups consuming chickpeas of any form or amount (e.g., whole, pureed, hummus, products containing chickpea flour/powder) | | | | | | | | | | | | |
| Control | | | Groups consuming a carbohydrate-containing meal without chickpeas (avCHO) | | | | | | | | | | | | |
| Certainty Assessment | | | | | | | | | N | | | |  | Effect | GRADE^*^ |
| Design | Risk of Bias ^1^ | | | Inconsistency ^2^ | | Indirectness ^3^ | Imprecision ^4^ | Other ^5^ | | Chickpea | avCHO | Studies |  | Absolute (95% CI) |  |
| **Glucose iAUC** | | | | | | | | | | | | | | | |
| RCT | Not Serious | | | Very Serious | | Not Serious | Not Serious | Publication bias:  undetected | | 300 | 293 | 15 | **SMD 1.13 lower**  (1.57 to 0.69 lower) | | ⨁⨁◯◯  Low |
| **Glucose C_max_** | | | | | | | | | | | | | | | |
| RCT | Not Serious | | | Very Serious | | Not Serious | Serious | Publication bias:  not evaluated | | 79 | 76 | 3 | **SMD 0.05 higher**  (0.59 lower to 0.68 higher) | | ⨁◯◯◯  Very Low |
| **Insulin iAUC** | | | | | | | | | | | | | | | |
| RCT | Not Serious | | | Not Serious | | Not Serious | Serious | Publication bias:  not evaluated | | 80 | 80 | 4 | **SMD 0.04 lower**  (0.41 lower to 0.33 higher) | | ⨁⨁◯◯  Low |
| * Grading of Recommendations, Assessment, Development and Evaluation (GRADE) Working Group grades of evidence | | | | | | | | | | | | | | | |
| ⨁⨁⨁⨁ | | High quality | | | Further research is very unlikely to change our confidence in the estimate of effect. | | | | | | | | | | |
| ⨁⨁⨁◯ | | Moderate quality | | | Further research is likely to have an important impact on our confidence in the estimate of effect and may change the estimate. | | | | | | | | | | |
| ⨁⨁◯◯ | | Low quality | | | Further research is very likely to have an important impact on our confidence in the estimate of effect and is likely to change the estimate. | | | | | | | | | | |
| ⨁◯◯◯ | | Very low quality | | | We are very uncertain about the estimate. | | | | | | | | | | |
| avCHO, available carbohydrate; CI, confidence interval; C_max_, peak glucose concentration; iAUC, incremental area under the curve | | | | | | | | | | | | | | | |

^1^ Evaluated by Cochrane risk-of-bias v2 (RoB 2) tool

^2^ Very serious in the case of substantial heterogeneity (I^2^ ≥ 50% and P < 0.10)

^3^ Directness to a priori PICOS parameters; Not serious if evidence sufficiently direct

^4^ Serious imprecision based on null effect with wide confidence intervals and few events

^5^ Publication bias analysis requires minimum of 10 studies

**Supplemental Figure 1.** Summary of risk of bias assessment evaluated using version 2 of the Cochrane risk-of-bias (RoB 2) tool.


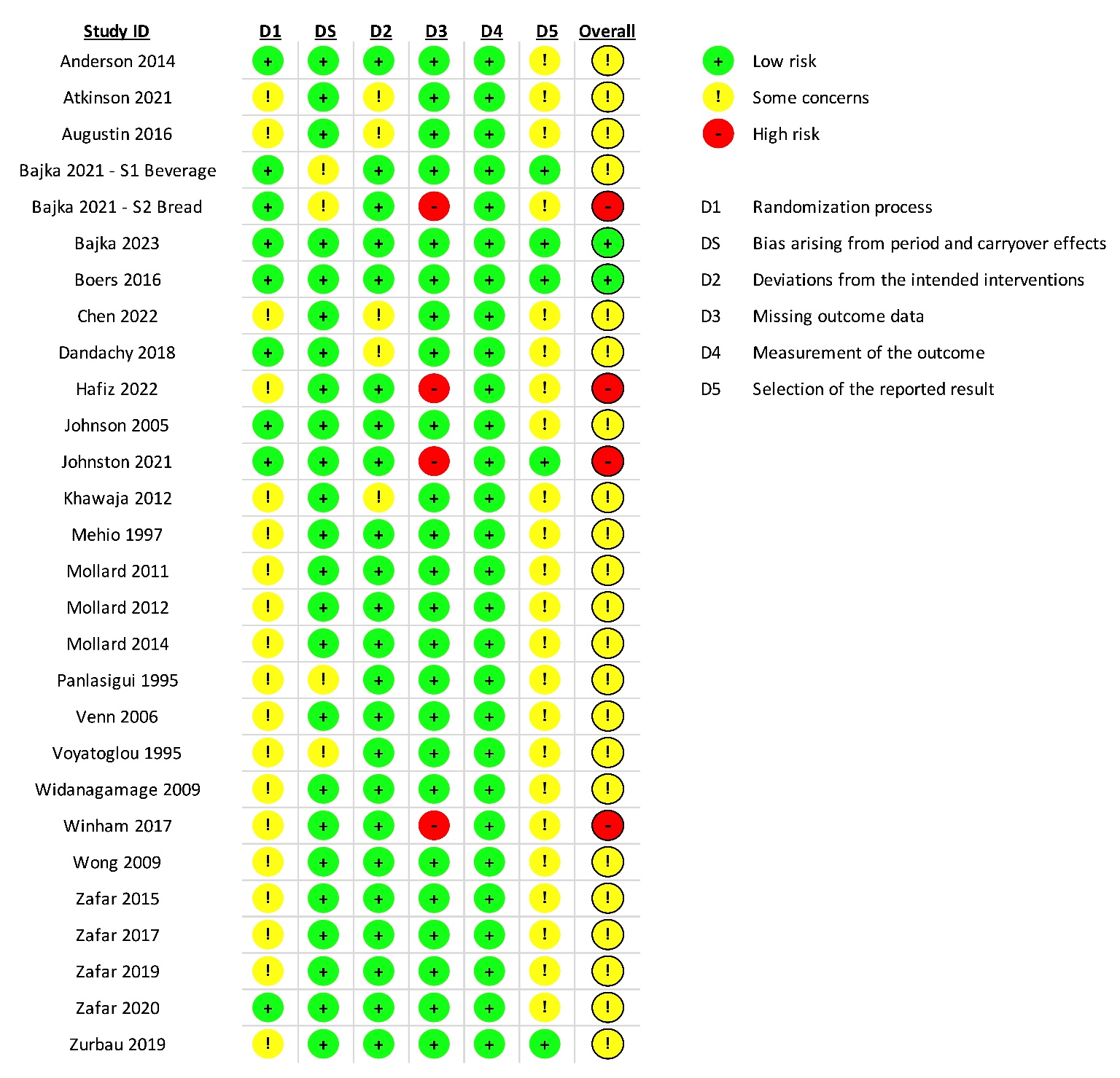


**Supplemental Figure 2. Publication bias for glucose area under the curve (iAUC).** Publication bias was evaluated through visual inspection of funnel plots for asymmetry and formal testing using Egger’s test

**
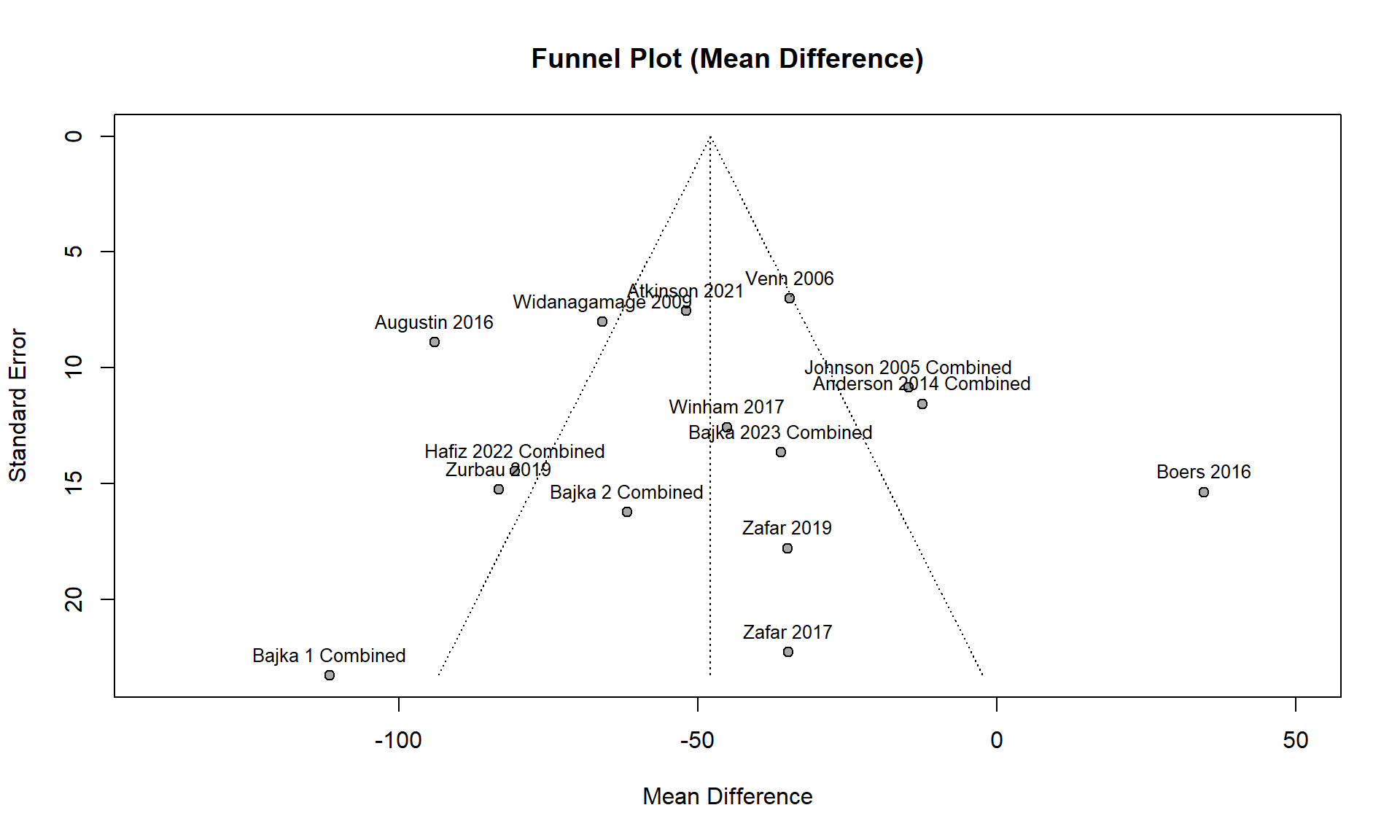
**
